# Benefits of Circulating Human Metabolites from Fish Cartilage Hydrolysate on Primary Human Dermal Fibroblasts, an Ex Vivo Clinical Investigation for Skin Health Applications

**DOI:** 10.3390/nu14235027

**Published:** 2022-11-25

**Authors:** Fabien Wauquier, Line Boutin-Wittrant, Elodie Bouvret, Julie Le Faouder, Véronique Roux, Nicolas Macian, Gisèle Pickering, Yohann Wittrant

**Affiliations:** 1 Clinic’n’Cell SAS, Faculty of Medicine and Pharmacy, TSA 50400, 28 Place Henri Dunant, CEDEX 1, 63001 Clermont-Ferrand, France; 2 Abyss Ingredients, 56850 Caudan, France; 3 CIC INSERM 1405, Plateforme d’Investigation Clinique CHU Gabriel Montpied, 58 Rue Montalembert, 63000 Clermont-Ferrand, France; 4 INRAE, UNH, 63009 Clermont-Ferrand, France; 5 Faculty of Medicine and Pharmacy, Clermont Auvergne University, UMR1019 of Human Nutrition, BP 10448, 63000 Clermont-Ferrand, France

**Keywords:** clinical trial, skin health, primary cells, human dermal fibroblasts, marine hydrolyzed collagen, chondroitin sulfate

## Abstract

Due to its significant exposure to stressful environmental factors, the skin undergoes a high remodeling rate over time, which alters not only its appearance but also its functionality. This alteration of the skin, namely photoaging, is characterized by dryness and a loss of elasticity that mainly originates from the dysregulation of dermal fibroblast activities. In order to overcome such tissue outcome, cosmetic products have evolved toward nutricosmetics, thus promoting beauty from within. Among bio-actives of interest, bio-peptides deriving from plant or animal sources may exert various biological activities beyond their nutritional value. However, studies remain mostly descriptive and the mode of action at the cellular level in clinic remains a concern. In a recent clinical trial, it was showed that supplementation with a fish cartilage hydrolysate (FCH) improved signs of chronological and photoaging-induced skin changes in healthy women. Here, using an original ex vivo clinical approach adapted to nutricosmetic purpose, we further demonstrated that this fish cartilage hydrolysate was absorbed and that the circulating metabolites produced in humans following FCH intake stimulate human dermal fibroblast growth, promote specific hyaluronan production, up-regulate elastin synthesis and inhibit MMP-1 and 3 expression along with the enhancement of TGF-β release. Altogether, these data provide clues on the mechanisms likely contributing to the beneficial impact of FCH on human skin functionality by supporting hydration, elasticity and limiting the expression of catabolic factors involved in photoaging onset.

## 1. Introduction

Aging is associated with an increased prevalence of chronic conditions due to the decline in the functionality of organs and tissues. The skin is the first biological barrier that interacts with our environment. It protects the body from external influences [1] and has a key role in the perception and the defense against physical, chemical or biological aggressions [2]. Due to its significant exposure to environmental factors, the skin undergoes a high remodeling rate over the time, which alters not only its appearance but also its functionality [3].

Known as photoaging, this alteration of the skin is characterized by dryness and a loss of elasticity that mainly originate from the dysregulation of dermal fibroblast activities [4]. At a molecular level, this translates into a decrease in both the quantity and the quality of the proteins and the glycosaminoglycans composing the extracellular matrix (ECM) [5]. To date, skin loses its capacity to hold water mainly due to a reduction in hyaluronic acid (hyaluronan-HA) synthesis [6]. Along with this, collagen and elastin undergo fragmentation and solar elastosis, which also characterizes cutaneous photoaging [7,8]. This collagenolysis and elastolysis are trigged by matrix metalloproteinases (MMPs) [4,9]. Interestingly, strategies targeting a reduction in MMPs expression were recently acknowledged for both preventing skin protein degradation and promoting photoaging skin cell repair [10]. Accordingly, pharmaceutical, cosmetics and food industries have promoted innovations to prevent, delay or minimize the signs of aging and preserve skin health [11]. Thus, over the last decade, the cosmetics industry has witnessed the rise of new concepts and strategies. One of the major developments in this field is that cosmetic products are no longer strictly limited to the preservation of physical appearance, but they may also be linked to significant health effects. The terms cosmeceutical and nutricosmetic have emerged to highlight this link and support a “beauty from within” [12].

Among bio-actives of interest, bio-peptides, usually short chains peptides of a few kDa deriving from plant or animal sources, may exert various biological activities beyond their nutritional values [13]. Collagen is an essential protein found in large amounts in human connective tissues, as well as the main component of bone, cartilage and skin [14]. As aforementioned, at the tissue level, aging leads to a loss of collagen and elastin [5]. As collagen can be easily sourced from different animal by-products, its potential pharmaceutical and cosmetic interest has attracted much attention.

Bio-peptides derived from the hydrolysis of collagen are acknowledged as active ingredients and thus naturally stand as relevant candidates for nutricosmetics. To date, oral intake of collagen peptides has already been linked to a beneficial effect on the osteoarticular system, either on bone or joint tissues [15,16,17]. In this context, the potential interest of this same FCH in the treatment of joint discomfort associated with osteoarthritis in rodents was recently demonstrated [18].

Along with collagen, chondroitin sulfate (CS) has also been reported for its nutritional value in preventing aged-related dysfunction of the osteoarticular tissues [19]. Thus, its importance in all connective tissues reinforces its relevance for the development of new cosmetic strategies. Chondroitin sulfate belongs to the glycoaminoglycans (GAGs) family. GAGs in general, and CS in particular, are essential components of the extracellular matrix of connective tissues. They play a key role in many biological processes such as the regulation of cell proliferation and differentiation and the control of the inflammatory process [20]. Moreover, its benefit on joint health partly relies on an inhibition of MMPs activities, which are also involved in skin aging [21].

From a “cosmetic” point of view, there is a recent growing literature that describes the beneficial effects of such ingredients on skin health at both preclinical and clinical levels [22]. For instance, hydrolyzed collagen was reported to protect skin against both natural aging [23] and aging related to UV exposure [24,25] through the preservation of tissue density [23,26] in different mouse models. At a clinical level, a Japanese study showed an improvement in trans-epidermal water loss and skin elasticity after 8 weeks of hydrolyzed collagen consumption at 10 g/day in volunteers aged 30 to 45 [27]. At the dose of 2.5 g/day, collagen intake increased the density of the dermis after 6 months of treatment in overweight or normally weighted women [28]. Recently, it was demonstrated that supplementation with this FCH reduced wrinkles in healthy women and increased dermis density and thickness [29]. Interestingly, most of the published clinical trials investigating the benefit of an oral collagen supplementation on skin health have been carried out in the last 10 years, supporting a major and timely interest related to the field. However, studies remain mostly descriptive and the mode of action at the cellular level in clinic remains unclear. Consequently, the literature supports the need to further investigate the molecular mechanisms involved at the cellular level in humans.

In this study, we demonstrated the biological activity of an FCH that presents a combination of hydrolyzed collagen peptides and CS and we aimed at both confirming the nutricosmetic potential of such an active ingredient and determining its mode of action in humans.

Using an ex vivo clinical approach developed at the French National Institute for Agronomic and Food Research (INRAE) [16,30,31,32,33], which considers the clinical digestive course of nutrients, we investigated the potential health benefits of the circulating metabolites following FCH ingestion in humans. In this light, we examined whether and how these human metabolites may influence major molecular protagonists responsible for skin hydration and remodeling.

## 2. Materials and Methods

### 2.1. Ethics Clinical Trial

This clinical investigation was conducted in conformity with the Declaration of Helsinki of 1975 (https://www.wma.net/what-we-do/medical-ethics/declaration-of-helsinki (accessed on 1 April 2021) revised in 2013. The clinical protocol was approved by the French Ethical Committee (2021-ND77 RIPH2 HPS/N° SI RIPH: 21.01436.000014/N° EudraCT/ID RCB: 2021-A01773-38/Comité de Protection des Personnes CPP Paris, Ile-de-France 1; approved 8 October 2021). The volunteers were told about the objectives and the potential risks of the investigation. They provided their written informed consent before being enrolled.

### 2.2. Study Product

The fish cartilage hydrolysate (CARTIDYSS^®^ NG, Abyss Ingredients) is an active food ingredient containing more than 65% of collagen peptides with a low molecular weight (under 3000 Da) and a minimum of 25% of CS. Each volunteer received 12 g of FCH, corresponding to 8.04 g of hydrolyzed collagen and 3.24 g of chondroitin sulfate.

### 2.3. Human Study Design and Pharmacokinetic of Absorption

A pool of 10 healthy men (age: 25.4 years old, +/−3.7; BMI: 23.6 kg/m^2^, +/−1.9; >60 kg; without drug treatment; and no distinction on ethnicity) volunteered for this study. They were verified for blood formulation, renal (urea and creatinine) and liver functions (aspartate aminotransferase (AST), alanine aminotransferase (ALT), gamma-glutamyltransferase (GGT) activities). Blood samples from all participants were obtained and collected in serum-separating tubes. Serum was teched, aliquoted and stored at the Centre d’Investigation Clinique de Clermont-Ferrand—Inserm 1405 (a specialized research department compliant with regulatory and ethical obligations and certified by the French government-NF S96900).

**Figure 1:**
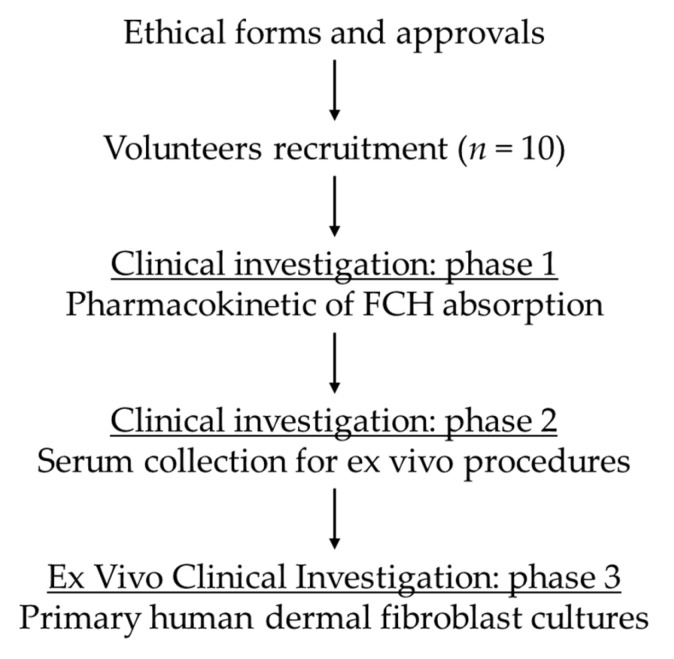
Clinical and ex vivo procedure flow.

The first step of the study aimed at determining FCH’s metabolites absorption peak. Ten healthy volunteers who fasted for 12 h were given 12 g of FCH. The dose was set according to validated preclinical [34,35] and clinical data [16,27,36,37,38]. Approximately 9 mL of venous blood was collected from the median cubital vein before the ingestion and every 20 min for 240 min after the ingestion. Serum was withdrawn from venous blood. Samples were immediately stored at −80 °C until further analyzed. Hydrolyzed collagen peptides and CS absorption profiles were evaluated by ELISA targeting hydroxyproline and CS, respectively. Once the absorption profile was determined, volunteers were called back for the collection of metabolites-enriched serum fractions. For this second clinical phase, 10 healthy volunteers fasted for 12 h and were then given 12 g of FCH. A total of 48 mL of venous blood was collected from the cubital vein before the ingestion for naive (baseline) serum collection. Then, at the maximum absorption peak, 48 mL of blood as well was collected for metabolites-enriched serum collection. Serum was stored at −80 °C until analysis (Figure 1).

### 2.4. Human Primary Dermal Fibroblasts (HDFs) Cultures

Human primary dermal fibroblasts from an adult donor were purchased from Sigma-Aldrich (Lyon, France, 106-05A). For maintenance, primary cells were cultured in Dulbecco’s Modified Eagle Medium (DMEM, Biowest, Nuaillé, France, L0066-500) supplemented with 10% fetal calf serum (FCS) (Invitrogen, Villebon-Sur-Yvette, France) and 1% penicillin/streptomycin (Life Technologies, Villebon-Sur-Yvette, France). Cells were cultured at 37 °C in an atmosphere of 5% CO_2_/95% air.

To analyze the effect of FCH, cells were seeded at 15,000 cells/cm^2^ either in 96- or 24-wells plates with 100 µL or 500 µL of culture medium, respectively, and allowed to grow for 3 days in maintenance media in order to reach 80–90% confluency. Cells were then incubated for 24 h in DMEM supplemented with 1% penicillin/streptomycin in the presence of 10% of human naive serum (H-NAIVE) or human serum enriched with circulating metabolites resulting from FCH ingestion (H-FCH) according to the Clinic’n’Cell protocol (DIRV INRA 18-0058).

### 2.5. Cell Viability

The ex vivo cell viability was determined using an XTT-based method (Cell Proliferation Kit II, Sigma-Aldrich, Saint-Quentin-Fallavier, France). Analyses were performed according to the supplier’s recommendations. Optical density was measured at 450 nm.

### 2.6. Hydroxyproline, Chondroitin Sulfate, Hyaluronan, Elastin and Transforming Growth Factor-β (TGF-β) Quantifications

Detection of circulating chondroitin sulfate and hydroxyproline was evaluated in serum using CS ELISA Kit (abx350001—Abbexa, Sugar Land, TX, USA) and hydroxyproline assay kit (MAK008—Sigma-Aldrich, Saint-Quentin-Fallavier, France), respectively. In HDFs, elastin, hyaluronan and TGF-β levels were evaluated in cell culture supernatant using Human Elastin ELISA Kit (ab239433—Abcam, Paris, France), Hyaluronan Quantikine ELISA Kit (DHYAL0—Bio-Techne, Minneapolis, MN, USA) and Human TGF beta 1 ELISA Kit (ab100647—Abcam, Paris, France), respectively, according to the manufacturer’s recommendations. Measurements were performed in quadruplicates for each sample condition of the ten volunteers.

### 2.7. Glycoaminoglycans Assay

GAGs production was determined in cell lysates using a dimethylmethylene blue (DMB) assay as previously described [39]. DMB solution was set at 46 mmol/L in a pH 3 adjusted buffer: 40 mmol/L NaCl, 40 mmol/L glycine. Sample concentrations were determined by mixing 25 µL of cell extract with 200 µL of DMB reagent. After a 30 min incubation, the OD was measured at 595 nm on an ELX808 IU spectrophotometer (BioTek Instruments, Winooski, VT, USA). GAGs content was compared to a standard curve of chondroitin sulfate (Sigma-Aldrich, Saint-Quentin-Fallavier, France). Results were reported to the total amount of proteins determined by BCA assay (Sigma-Aldrich, Saint-Quentin-Fallavier, France) and expressed as CS equivalents. Measurements were *n =* 4 for each sample of the ten volunteers.

### 2.8. Real-Time RT-qPCR

mRNAs from HDFs were isolated using TRIzol™ Reagent (Ambion—Life Technologies, Villebon-Sur-Yvette, France) according to the supplier’s recommendations. COL1A1 (Collagen Type I Alpha 1), MMP1 (Matrix Metalloproteinase-1) and MMP3 (Matrix metalloproteinase-3) mRNA expression levels were measured by RT-qPCR (PowerUp SYBRgreen, Applied Biosystems, Waltham, MA, USA). β-Actine was used as a housekeeping gene. Primers were designed as follows: COL1A1-F: 3′-TTC TGT ACG CAG GTG ATT GG-5′; COL1A1-R: 3′-GAC ATG TTC AGC TTT GTG GAC-5′; MMP1-F: 3′-GCC AAA GGA GCT GTA GAT GTC-5′; MMP1-R: 3′-GAC AGA GAT GAA GTC CGG TTT-5′; MMP3-F: 3′-TGA GTG AGT GAT AGA GTG GGT-5′; MMP3-R: 3′-TGA ACA ATG GAC AAA GGA TAC AAC-5′; ACTβ-F: 3′-ATT GGC AAT GAG CGG TTC-5′; ACTβ-R: 3′-GGA TGC CAC AGG ACT CCA-5′.

### 2.9. Statistics

Prism V.9.4.1 (GraphPad Software, San Diego, CA, USA) was used to run statistic tests and set figures. The following statistic plan was applied: Gaussian distribution was evaluated according to Shapiro–Wilk normality test. In case of non-normal distribution, a Kruskal–Wallis nonparametric test was used followed by Dunn test for post hoc comparison. When normal distribution and equal variance were assumed, measures were subjected to one-way ANOVA with Tukey’s test for multiple comparisons. Values are presented as the means ± SD unless specified otherwise. The differences were considered statistically significant with * for *p* < 0.05, ** for *p* < 0.01, *** for *p* < 0.001 and **** for *p* < 0.0001.

## 3. Results

### 3.1. Kinetic Profile of FCH Absorption

The clinical study was designed in two phases. The first phase aimed at characterizing the kinetics of the apparition of the metabolites following FCH ingestion in order to both (1) evidence the human absorption of the ingredient and (2) determine the time frame of the absorption peak for the second phase. The second phase was dedicated to collect both naive and enriched sera, before ingestion and at the absorption peak, respectively. Both naive and enriched human sera were subjected to ex vivo investigations to evaluate the influence of human metabolites resulting from the consumption of FCH on the function of human dermal fibroblasts.

In order to reach this aim we tracked the blood concentration of both hydroxyproline and chondroitin sulfate. Fasted volunteers received 12 g of FCH and the absorption profile was monitored on a 240 min period of time.

**Figure 2:**
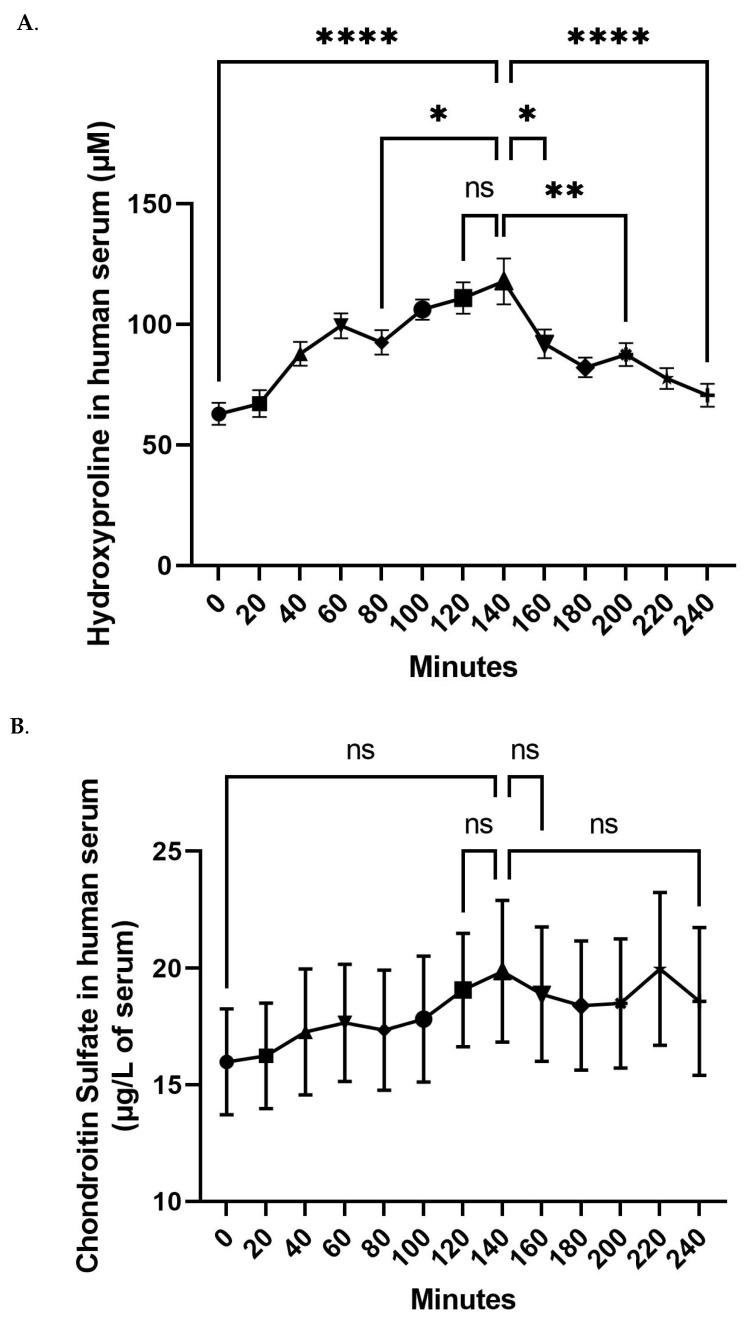
Evolution of the concentration of the circulating hydroxyproline (**A**) and chondroitin sulfate (**B**) in blood. Values are presented as the means ± SEM. The differences were considered statistically significant at *p* < 0.05 with * for *p* < 0.05; ** for *p* < 0.01; **** for *p* < 0.0001 and ns for *p* > 0.05.

The analyses showed that following the ingestion of FCH, circulating concentrations of hydroxyproline continuously increase to reach a maximum of 117.7 µM at 140 min post-ingestion (+87.5% compared to the basal level), before returning to an almost basal level by the end of the kinetics (Figure 2A). The maximum serum concentration for CS was also observed at 140 min post-ingestion (19.9 ng/mL; +24.2% compared to the basal level). However, in contrast to hydroxyproline data, none of the calculated concentrations of circulating CS significantly differed from each other. According to the circulating hydroxyproline concentration profiles, the collection of enriched serum with FCH metabolites for the second phase of the clinical protocol was set at 140 min post-ingestion.

### 3.2. Validation of the Cell Model Ex Vivo Procedures

The ultimate goal of this ex vivo investigation was to evaluate the impact of human circulating metabolites following FCH ingestion on primary human dermal fibroblasts.

**Figure 3:**
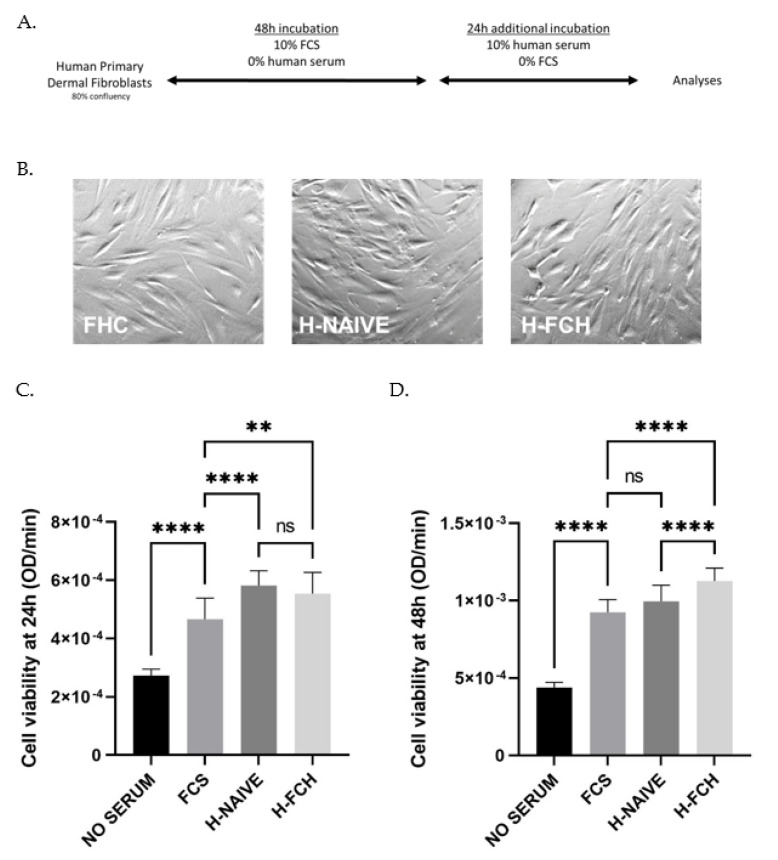
Primary human dermal fibroblasts (adult) subjected to ex vivo procedures (**A**). Regular fibroblastic shape upon human serum incubation (**B**). Cell viability measured with an XTT-based assay upon either FCS or human serum incubation (H-NAIVE for human naive serum and H-FCH for human serum enriched with circulating FCH metabolites) for 24 h and 48 h (**C**,**D**). Measures were performed in quadruplicates per condition/volunteer (*n =* 10 volunteers). Values are presented as the means ± SD. The differences were considered statistically significant at *p* < 0.05 with ** for *p* < 0.01; **** for *p* < 0.0001 and ns for *p* > 0.05.

Cell culture assays were set as shown in Figure 3A. In order to ensure the physiological relevance of our ex vivo approach, we verified the influence of the different human sera on cell behavior and cell growth (Figure 3A). In the presence of human serum, the cells undertook a regular fibroblastic shape (Figure 3B). Using an XTT-based method, we compared cell growth to a regular FCS incubation. As expected, cell growth drastically slowed down in serum-free cultures, while the cells kept growing in the presence of FCS 10% (Figure 3C). Naive or enriched human sera processed according to the Clinic’n’Cell methodology (DIRV#18-0058; see the patents section) did not impair the cells’ behavior as compared to conventional fetal calf serum, and rather enhanced cell growth and viability, supporting further investigations.

### 3.3. FCH Modulates GAGs and HA Synthesis

**Figure 4:**
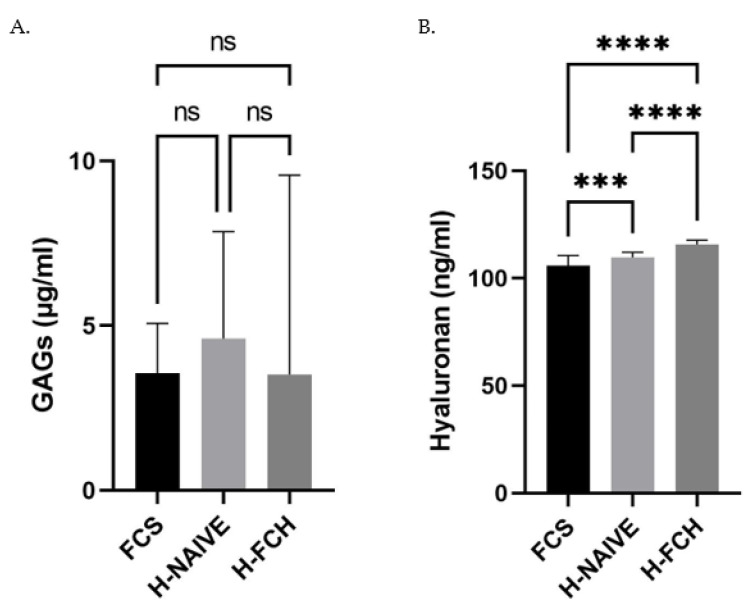
Primary human dermal fibroblasts (adult) were incubated in the presence of either fetal calf serum (FCS) or human naive serum (H-NAIVE) or human serum enriched with metabolites deriving from FCH ingestion (H-FCH). Glycosaminoglycans (GAGs) (**A**) and hyaluronan (HA) (**B**) production. Measures were performed in quadruplicates per condition/volunteer (*n =* 10 volunteers). Values are presented as the means ± SD. The differences were considered statistically significant at *p* < 0.05 with *** for *p* < 0.001; **** for *p* < 0.0001 and ns for *p* > 0.05.

Glycosaminoglycans (GAGs) are key factors responsible for skin hydration and are therefore major contributors to the maintenance of skin health and function. Thus, we verified the influence of the human FCH metabolites on GAGs production by primary human dermal fibroblasts. Neither the naive nor the enriched human serum had any significant effect on this parameter (Figure 4A). In contrast, hyaluronan synthesis was slightly but significantly increased by the human serum and further stimulated in the presence of FCH metabolites (Figure 4B). Taken together, these data suggest a targeted effect of FCH on specific GAGs involved in skin hydration rather than a mere global effect.

### 3.4. FCH Modulates Collagen and Elastin in the Extracellular Matrix

**Figure 5:**
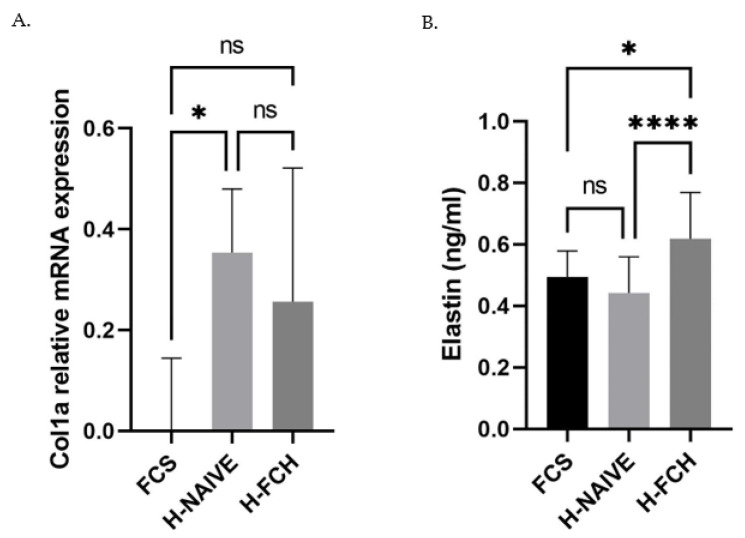
Primary human dermal fibroblasts (adult) were incubated in the presence of either fetal calf serum or human naive serum (H-NAIVE) or human serum enriched with metabolites deriving from FCH ingestion (H-FCH). Collagen 1a mRNA expression (**A**) and elastin protein concentration (**B**). Measures were performed in quadruplicates per condition/volunteer (*n =* 10 volunteers). Values are presented as the means ± SD. The differences were considered statistically significant at *p* < 0.05 with * for *p* < 0.05; **** for *p* < 0.0001 and ns for *p* > 0.05.

Along with hydration, we evaluated the impact of circulating metabolites from FCH on the capacity of HDFs to promote collagen and elastin synthesis, two main compounds of the extracellular matrix of skin tissues, whereas for naive human serum-enhanced collagen mRNA expression, when compared to FCS, the enrichment in FCH metabolites had no significant influence (Figure 5A). In contrast to collagen expression, there was no difference between FCS and human naive serum influence on elastin protein level, while the presence of FCH metabolites potently increased its production by 40% (Figure 5B).

### 3.5. FCH Modulates Proteolytic Enzymes Expression Involved in ECM Degradation

**Figure 6:**
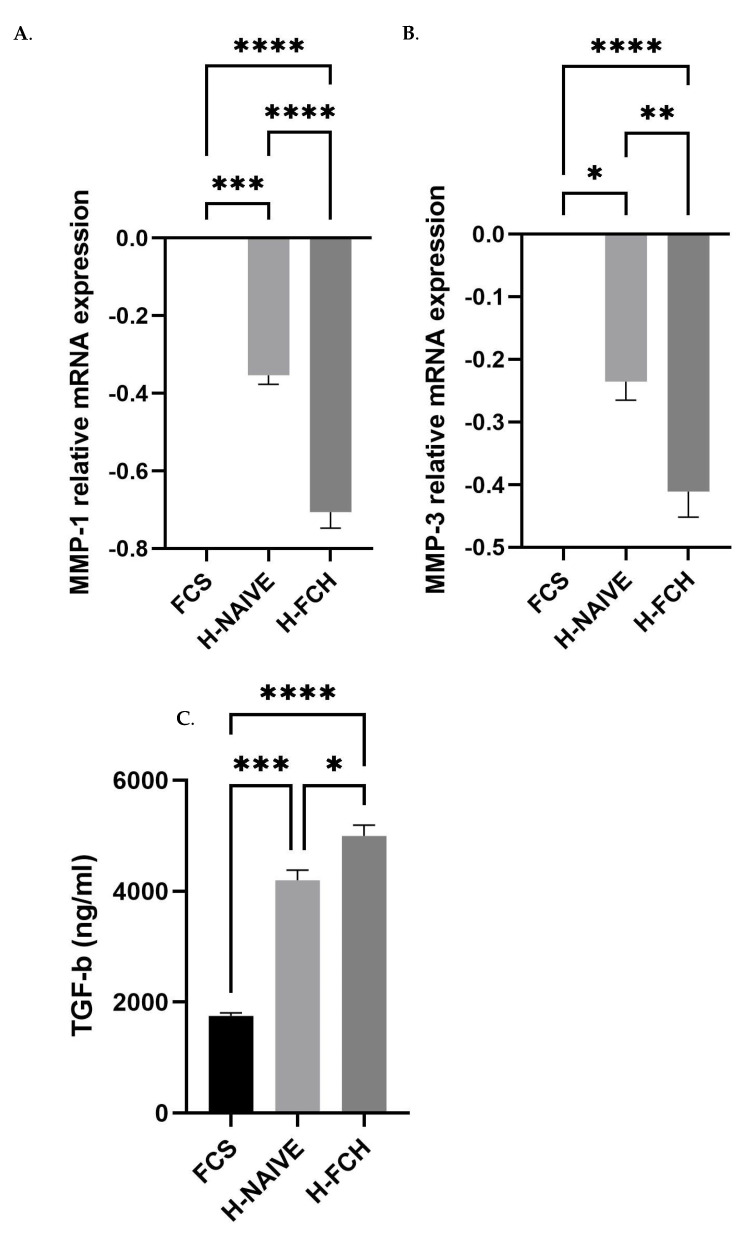
Primary human dermal fibroblasts (HDFs—adult) were incubated in the presence of either fetal calf serum or human naive serum (H-NAIVE) or human serum enriched with metabolites deriving from FCH ingestion (H-FCH). MMP1 and MMP3 mRNA relative expression (**A**,**B**). TGF-β release by HDFs (**C**). Measures were performed in quadruplicates per condition/volunteer (*n =* 10 volunteers). Values are presented as the means ± SEM. The differences were considered statistically significant at *p* < 0.05 with * for *p* < 0.05; ** for *p* < 0.01; *** for *p* < 0.001 and **** for *p* < 0.0001.

Besides anabolism features, the aim of this study was also to evaluate the ability of circulating FCH metabolites to limit the expression by HDFs of degradation factors responsible for photoaging-related skin alteration. The human naive serum was prone to inhibit both MMP1 and MMP3 expression compared to FCS (Figure 6A,B). Of great interest was that the presence of FCH metabolites significantly and potently further inhibited the expression of MMP1 (−50.2%) and MMP3 (−30.9%) when compared to naive human serum. Since MMP regulation may occur through TGF-β signaling, we questioned its release by HDFs upon incubation with FCH metabolites. Interestingly, the TGF-β concentration in culture supernatants was stimulated by the human serum and further up-regulated upon enrichment with FCH human metabolites (Figure 6C).

## 4. Discussion

In this new clinical investigation, we first aim to ensure that the FCH exposure was safe and physiologically sound for our nutricosmetic purposes. Thus, the dose used for our ex vivo protocol was compared to the literature data. FCH is mainly composed of hydrolyzed collagen (HC) and CS. Animal toxicity studies have demonstrated the absence of deleterious effects for such compounds. For CS, a single dose of 2 g/kg has been tested in rodents without any adverse effects [35]. The LD50 for oral intake of HC in rodents is greater than 10 g/kg [34]. From a nutraceutical point of view, effective doses of HC for skin health benefits in mouse models vary between 200 and 800 mg/kg [23,26]. These doses would roughly correspond to a range from 2 g to 8 g in humans. The dose of HC alone or combined with GAGs used for clinical investigations ranges from 200 mg/day to 10 g/day (mostly 1 g/day) for a period of time generally encompassing 2 to 6 months [27,28,40,41]. In a very recent ex vivo clinical study, volunteers received 25 g of HC from bovine origin in a single shot without any side effects [16]. Here, volunteers were given 12 g of FCH, half of the aforementioned dose, and none of them reported any discomfort. Additionally, in this study, the human sera used for cell culture investigations were diluted ten times. Thus, we set the single exposure of FCH to the dose of 12 g for more accurate nutritional consistency.

Regarding the absorption of FCH, the literature lacks relevant kinetics data; thus, this study also aimed at evidencing and characterizing the bioavailability of FCH in humans in order to both reinforce the clinical value of our previous results on skin health in middle-aged women [29] and to move forward on the mechanism of action at the cellular level.

To date, two previous clinical studies from the same team and from the 2000s showed that the ingestion of Chondrosulf^®^, an ingredient composed of CS from bovine origin, led to an absorption peak around 2 h post-ingestion [37] with a return to a basal level at around 6 h [38]. Consistently, even non-significantly, we found that circulating CS concentration reached a maximum at 140 min and then decreased almost to baseline after 4 h. The administrated dose was similar to ours. Volpi et al. used 4 g of Chondrosulf^®^ (bovine origin) while we used 12 g of FCH with 3.24 g of CS from marine origin. In a more recent study, Passov et al. focused on basal concentrations of circulating CS. Using the same ELISA approach as ours, they showed that the basal level of CS in plasma was about 12.7 ng/mL, and therefore further supports the reliability of our data [42].

In this study, HC’s absorption peaked at 140 min. Consistent with our observations, orally ingested collagen was proven to undergo degradation into oligopeptides that can be detected in blood two hours after ingestion [43], and hydroxyproline blood concentration was demonstrated to range in µM (nmol/mL) [44,45]. Interestingly, Taga et al. reported that following the ingestion of 25 g of fish gelatin hydrolysate, the plasma concentrations reached about 100 nmol/mL for “total hydroxyproline-containing peptides” between 1 and 2 h and returned to the initial level after 6 h [46]. These data nicely parallel with ours, since we observed, for circulating hydroxyproline, a Tmax at 140 min and a Cmax of 117.7 nmol/mL. Moreover, it is worth noting that here we reached a similar Cmax with half the dose ingested in the Taga’s study, thus supporting the fact that the FCH used in this study is well absorbed. In this light, the size of the bio-peptides may matter. In vitro, in a model of enterocyte-like Caco-2 cells, HC with low molecular weight was previously shown to have a greater transport efficiency across the cell monolayer than the unhydrolyzed collagen control [47]. Focusing on fish products, a smaller size of fish collagen peptides was associated with higher efficient transport across a Caco-2 monolayer through a paracellular-dependent pathway [48]. According to its hydrolysis process, FCH is mainly composed of peptides of less than 3 kDa, which may likely contribute to a better assimilation, and subsequently to the observed biological activity.

Skin aging is characterized by dryness and both collagen and elastin networks fragmentation in the dermis. Therefore, hydration and tissue densification have become a major concern for nutricosmetic strategies. In this light, water-binding compounds such as GAGs and most particularly HA have attracted much attention. In vitro, histological slides of skin explants incubated directly with HC evidenced an increased GAGs level in the basal epidermis [49]. In two randomized, placebo-controlled clinical trials from Asserin et al., oral supplementation with 10 g of HC for 12 weeks improved the skin moisture level by 12%, as measured by conductance. Here, the incubation of HDFs with human metabolites from FCH slightly but significantly promoted HA synthesis but not global GAGs production, suggesting a specific biological effect rather than a mere influence on GAGs production.

To date, the effects of chondroitin polysulfate (CPS), a semi-synthetic oversulfated CS, on the metabolism of extracellular matrix in HDFs demonstrate that CPS accelerated the production of GAGs but did not modulate the production of collagen. Moreover, more than 80% of total synthesized GAGs were found to be HA [50]. These data greatly echo with ours on the modulation of HA, GAGs and collagen and further highlight the relevance of CS in combination with HC in mediating the skin health benefits of FCH.

Moreover, HA has been reported to facilitate collagen and elastin interaction and, therefore, to promote a proper tissue matrix configuration. In contrast, during aging, loss of HA may contribute to the disorganization of collagen and elastin fibers [51]. In a double-blind, placebo-controlled study, bioactive collagen peptide VERISOL^®^ given at the dose of 2.5 g daily for 8 weeks enhanced the skin content of both procollagen type I (65%) and elastin (18%) [52]. In vitro and histological investigations on skin explants cultured with HC were less conclusive [49]. Here, we did not find any impact of FCH metabolites on collagen expression but a strong and significant up-regulation on elastin synthesis by HDFs. To date, during late stages of skin healing, CS synthesis is associated with selective areas in which extra-cellular matrix actively remodels. Such areas were positively associated with elastin de novo synthesis [53], providing clues on the positive role of CS on skin homeostasis.

As aforementioned, skin aging and most notably photoaging is associated with protein degradation in the skin’s ECM [49]. Thus, nutraceutical strategies have focused on the reduction in factors responsible for such alterations [54]. Here, we found that FCH metabolites potently down-regulate MMP-1 and MMP-3 expression compared to naive human serum. Remarkably, such a down-regulation of MMP-1 and MMP-3 may have contributed to the augmentation of elastin level observed in this study. In this light, it is worth noting that CS benefits on cartilage matrix and on joint health in general also rely on an inhibition of MMPs activities [21]. Taken together, these data strongly support the fact that the health benefit of FCH occurs both by stimulating skin hydration and elastin synthesis while reducing proteolytic protagonists. As observed in the last figure of the manuscript, the inhibition of MMP-1 and -3 expressions parallels with the rise in TGF-β release by HDFs following incubation with FCH metabolites. Consistent with our data, the activation of the TGF-β/Smad pathway was recently reported to down-regulate MMP-1 and MMP-3 protein expression and prevent collagen degradation, thus promoting photoaging skin cell repair [10]. In this study, we provide details, at a human level, on the role and the mode of action of FCH metabolites and the importance of the combination of HC and CS on the preservation of HDFs activity. To the best of our knowledge, such relationships have not been reported yet on skin health.

We used primary HDFs to keep the cellular model consistent with the clinical dimension of this investigation. HDFs are key cellular players in regulating skin health and allowed for us to investigate the mode of action of FCH in preserving skin tissues from age-related cell damages. However, additional investigations on human primary keratinocytes may further provide clues on the possible influence of FCH on the keratinocytes/fibroblast cross-talks and their positive impact on skin health.

Collagenolysis and elastolysis by MMPs occur in photoaging and wound healing as well [9]. Interestingly, GAGs in general and collagen hydrolysates in particular are widely studied for their facilitating role in healing processes. Indeed, they may support skin tissue engineering [55,56] by promoting both the proliferation and migration of primary human fibroblasts from the dermis [57] and the differentiation and the migration of human keratinocytes as well [58]. Moreover, TGF-β is also involved in fibroblast proliferation and keratinocyte differentiation in wound healing [59,60]. Consequently, investigations on FCH influence on healing processes are ongoing in our laboratory.

## 5. Conclusions

It was recently shown that the supplementation of middle-aged healthy women with this FCH led to both a significant reduction in wrinkles and an increase in dermis density [29]. In this clinical ex vivo approach, considering the physiological processes of nutrients along the digestive track, we demonstrated that this FCH was absorbed and that the circulating metabolites produced in humans following FCH intake stimulate human dermal fibroblast growth, promote specific hyaluronan production, up-regulate elastin synthesis and inhibit MMP-1 and 3 expressions along with the enhancement of TGF-β release. Altogether, these data provide valuable new insights on the mechanisms likely contributing to the beneficial impact of FCH on human skin functionality by supporting hydration, elasticity and limiting the expression of catabolic factors involved in photoaging onset.

## 6. Patents

The human ex vivo methodology used in this study has been registered as a written invention disclosure by the French National Institute for Agronomic, Food and Environment Research (INRAE) (DIRV#18-0058). Clinic’n’Cell^®^ has been registered as a brand [16,30,31,32,33].

## Data Availability

The data presented in this study are available upon request from the corresponding author. The data are not publicly available due to ethical restrictions.
